# RETRACTION: Analysis of Risk Factors Affecting Wound Healing and Wound Infection After Meningioma Resection

**DOI:** 10.1111/iwj.70625

**Published:** 2025-04-21

## Abstract

**RETRACTION:** WangJ. , ShouJ. , GaoH. , WangB. , DingP. , and YangP. , “Analysis of Risk Factors Affecting Wound Healing and Wound Infection After Meningioma Resection,” International Wound Journal 21, no. 4 (2024): e14870, 10.1111/iwj.14870.38629599 PMC11022305

The above article, published online on 17 April 2024, in Wiley Online Library (http://onlinelibrary.wiley.com/), has been retracted by agreement between the journal Editor in Chief, Professor Keith Harding; and John Wiley & Sons Ltd. Following an investigation by the publisher, all parties have concluded that this article was accepted solely on the basis of a compromised peer review process. The editors have therefore decided to retract the article. The authors did not indicate their agreement with the retraction.

**RETRACTION:** J. Wang , J. Shou , H. Gao , B. Wang , P. Ding , and P. Yang , “Analysis of Risk Factors Affecting Wound Healing and Wound Infection After Meningioma Resection,” International Wound Journal 21, no. 4 (2024): e14870, 10.1111/iwj.14870.38629599 PMC11022305

The above article, published online on 17 April 2024, in Wiley Online Library (http://onlinelibrary.wiley.com/), has been retracted by agreement between the journal Editor in Chief, Professor Keith Harding; and John Wiley & Sons Ltd. Following an investigation by the publisher, all parties have concluded that this article was accepted solely on the basis of a compromised peer review process. The editors have therefore decided to retract the article. The authors did not indicate their agreement with the retraction.

